# Association Between Laboratory Metrics and Mortality After Major Lower Extremity Amputation in Peripheral Artery Disease Patients

**DOI:** 10.3390/jcm14082640

**Published:** 2025-04-11

**Authors:** Amun Georg Hofmann, Emanuel Greistorfer, Fadi Taher, Afshin Assadian, Maria Elisabeth Leinweber

**Affiliations:** Department of Vascular and Endovascular Surgery, Klinik Ottakring, Montleartstrasse 37, 1160 Vienna, Austria

**Keywords:** major amputation, peripheral artery disease, lower extremity, mortality

## Abstract

**Introduction**: Apart from their high burden of disease, major amputations, especially due to macro- and microangiopathic malperfusion, persist to inflict a relevant socioeconomic impact in most geographic regions. It has been repeatedly shown that lower extremity amputations are associated with impaired post-operative survival. In the present study, we investigated whether metrics derived from routine laboratory studies after amputation are associated with post-operative survival. **Methods**: In this retrospective single-center analysis, 244 patients undergoing lower extremity amputation between 2012 and 2016 were included. Serum hemoglobin and leukocyte counts of the first 21 post-operative days as well as derived metrics were analyzed in addition to clinical and demographic variables. Kaplan–Meier estimates and adjusted Cox regressions were fitted including relevant parameters. **Results**: In summary, 71.3% of patients underwent transtibial and 28.7% transfemoral amputations. The most frequent post-operative complications were wound-related (43.0%). Long-term survival analyses showed that advanced age and higher ASA class were significantly associated with reduced post-operative survival, while no significant survival differences were observed based on sex, smoking history, or type of amputation. Laboratory parameter analysis showed impaired peri-operative outcomes in patients with elevated leukocyte counts, with leukocyte-derived metrics showing significant associations with long-term survival after adjustment for age and ASA class. **Conclusions**: This study highlights the potential of routine laboratory parameter-derived metrics in predicting mortality after major lower extremity amputations in PAD patients.

## 1. Introduction

Various conditions can result in or require lower extremity amputations, including trauma or malignancies, with peripheral arterial disease (PAD) and diabetes representing the leading causes globally [1,2,3]. More than 200 million patients are estimated to be affected by PAD, and major amputations continue to be a frequent surgical intervention in cases where revascularization failed or is no longer considered feasible [3]. The implications and sequelae of major amputation are severe, encompassing physical, psychological, and economic aspects, as well as high post-operative mortality rates [4,5,6]. An analysis based on health insurance claim data focusing on hospital mortality rates following major amputation in Europe has shown that it ranges between 6.1% and 20.8%, with Austria falling in the middle at 14.3% [2]. It is well reported by now that mortality after major amputation is high, with reported proportions of 47.9% at one year and 70.6% at three years, estimates that are comparable to many types of malignancies [7].

Associations between serological biomarkers and mortality are well investigated and have been repeatedly shown to improve mortality predictions beyond clinical characteristics ranging from limited samples in specific pathological conditions to large epidemiological cohorts. Previous investigations cover a wide array of potential biomarkers, from routinely collected laboratory parameters such as lipoproteins [8] or serum levels of liver enzymes [9] to biomarkers collected with advanced biochemical assays outside of standard clinical practice [10,11]. In particular, post-operative survival is a frequently investigated matter, and markers of inflammation such as C-reactive protein have been shown to be associated with mortality in a variety of underlying conditions [12,13,14,15]. While there is large variation between study designs, they frequently conclude that markers of inflammation are an independent risk factor for post-operative mortality.

In a recent analysis of data concerning survival rates in dialysis patients, it was shown that the area outside the suggested target range for hemoglobin (HGB) is significantly associated with survival [16]. The authors therefore not only investigated pure HGB values as a quantitative variable or anemia as a binary outcome but quantified the excursion outside of the target range as an area under the curve. This retrospective study, utilizing data from patient medical records, as well as population-based data from the National Death Registry, aims to investigate whether HGB and leukocyte counts as well as related metrics such as the area outside the reference are associated with mortality after lower extremity amputation in PAD patients.

## 2. Methods

### 2.1. IRB Approval

Ethical approval was obtained prior to the commencement of the study from the Ethics Committee of the City of Vienna (approval number: EK 22-214-VK).

### 2.2. Study Design and Patient Data

This study was conducted as a retrospective single-center analysis. Patient data were extracted from electronic medical records including demographics, comorbidities, and treatment characteristics. The study included all patients of 18 years or older who underwent major amputation due to microangiopathic disease or PAD between 2012 and 2016. In total, 244 patients met the inclusion criteria. Mortality data were obtained from the Austrian National Death Registry and linked with data extracted at the study center with the most recently available data (cutoff date: 31 December 2023). Censored patients were therefore either alive at this date or lost to follow-up, for example, in cases of relocation to a different country. Plausibility checks were manually conducted on the collected data to ensure accuracy and consistency.

### 2.3. Laboratory Data

Laboratory data extracted from the electronic medical records included hemoglobin (HGB) [g/dL] and leukocyte count [G/L] starting from the pre-operative laboratory study (either on the day of surgery or the day before) until the 21st post-operative day. In cases where more than one laboratory study was available on a given day, results from the study with higher or lower values were extracted for leukocytes and HGB, respectively. Since a high leukocyte count and a low HGB value were considered relevant for the outcome of interest, maximum and minimum values, respectively, were calculated for each patient. Furthermore, mean over the observational period, variance, and the area outside the reference values were calculated for each patient. The area outside the reference was calculated as the area under the curve above the value of 10 for leukocytes and below 12.5 for HGB. This was done by fitting a spline to connect measured laboratory values and calculating the area between the resulting curve and boundary set by the reference threshold.

### 2.4. Analysis

All statistical analyses were conducted using R (v. 4.1.3, R Foundation for Statistical Computing, Vienna, Austria). Descriptive statistics were used to display study population characteristics. Categorical variables are shown as frequencies and proportions. Continuous variables are shown as median and Q1–Q3. Group means were compared by *t*-tests and categorical variables were compared by chi-square tests between groups. This analysis followed a multi-step analytical workflow. First, crude survival probabilities were estimated using Kaplan–Meier curves and stratified by various subgroups, utilizing data from the National Death Registry for each patient in the dataset. Second, the included laboratory parameters and related metrics were investigated for associations with peri-operative outcomes to identify potential confounding effects. Third, Cox regressions were conducted to adjust for relevant confounders and identify parameters associated with survival. In the sensitivity analysis, peri-operative days within the first 30 days were excluded to isolate mid- and long-term survivors. For standard hypothesis tests, a *p* value < 0.05 was considered statistically significant, whereas for predictors in the regression model, a *p*-value < 0.1 was considered the threshold for elimination in the models.

## 3. Results

### 3.1. Patient Demographics

In summary, 244 patients underwent major amputation between 2012 and 2016, with 71.3% undergoing transtibial amputation and 28.7% undergoing transfemoral amputation. The majority of patients were male (65.6%), with a median age of 72 years (Q1–Q3: 65–79 years). At the time of hospital admission, 18.0% of patients were diagnosed with critical limb ischemia at Rutherford stage 4, while 20.9% presented with Rutherford stage 5, and 50.4% with Rutherford stage 6. In 10.6% of patients, the medical records did not provide sufficient information to distinguish between Rutherford stage 5 or 6. In the present study population, 25 patients (10.2%) had a recorded major amputation of the contralateral leg in their medical history. Ten patients (4.1%) were on chronic hemodialysis at the time of surgery. The main patient characteristics are depicted in Table 1.

### 3.2. Major Amputation and Post-Operative Outcomes

Most patients (67.6%) underwent surgery under general anesthesia. Post-operative complications, including both minor and major, were documented in 127 patients. Wound complications were the most frequent, including infections, hematomas, or tissue necrosis, occurring in 43.0% of all patients. Regarding non-surgical complications, 26 patients (10.7%) experienced cardiac complications including cardiac decompensation, myocardial infarction, or cardiac arrhythmia. Overall, 19.6% required surgical re-intervention with further proximal bone resection. The median length of hospital stay was 54 days (Q1–Q3: 35–86). However, patients with a wound complication had a significantly longer stay compared to patients without wound complications (79.6 vs. 50.3 days, *p*-value < 0.001). There was no statistically significant difference regarding wound complications in diabetic and non-diabetic patients (41.7% vs. 43.9%, *p*-value > 0.05).

### 3.3. Survival Analysis

**Figure 1:**
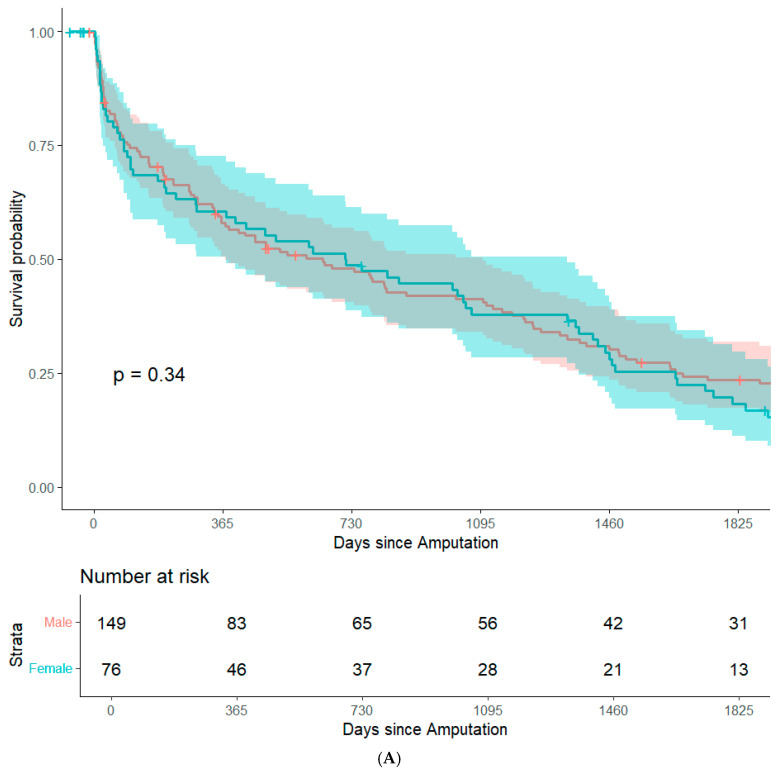

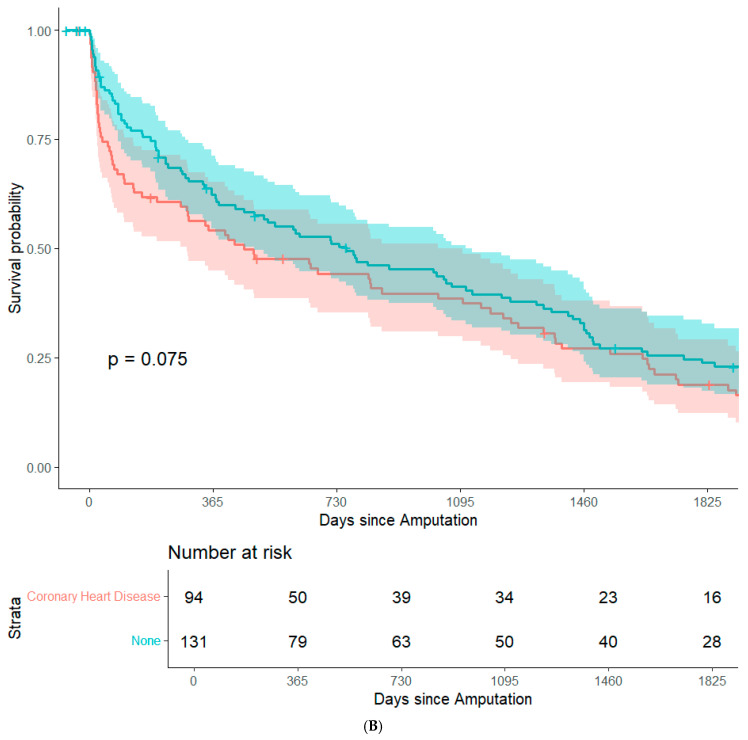

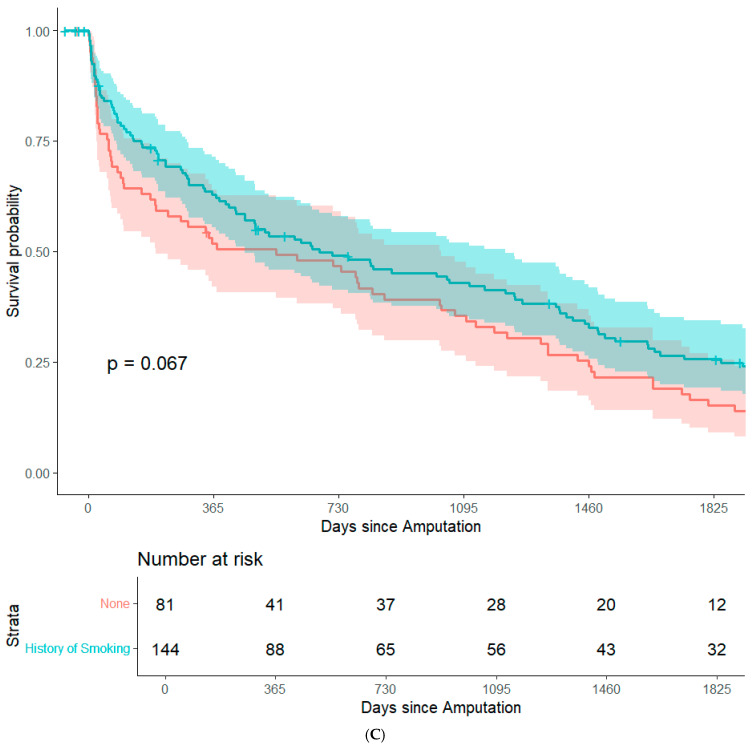

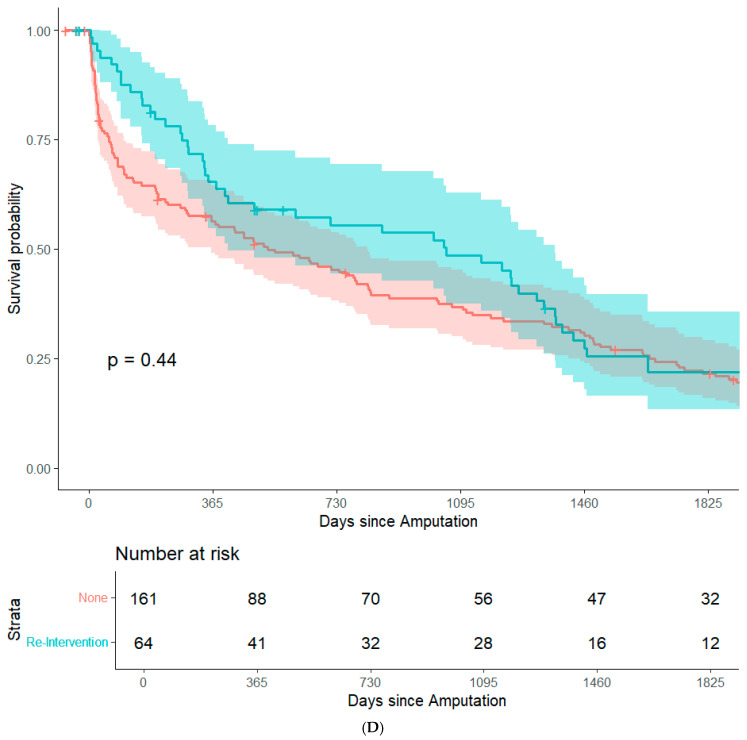

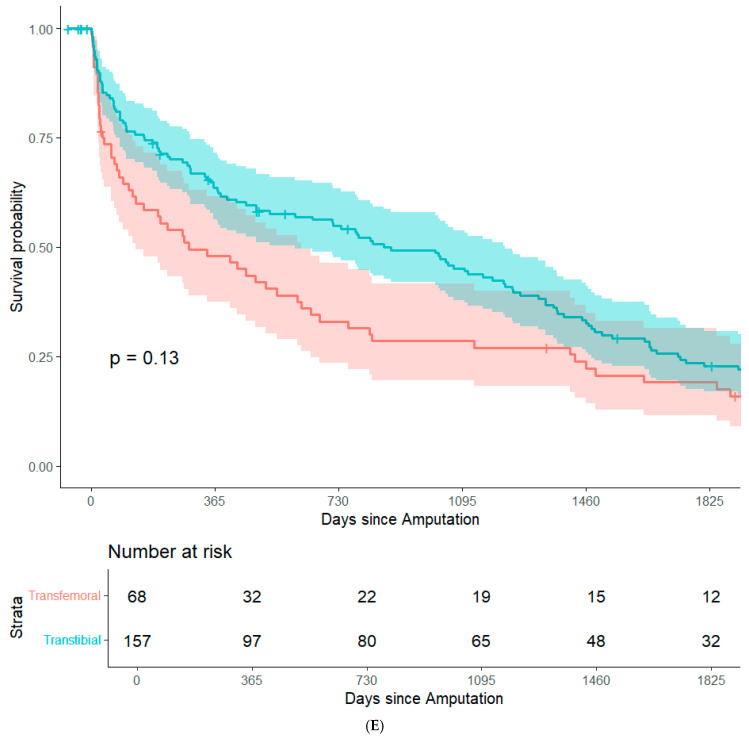

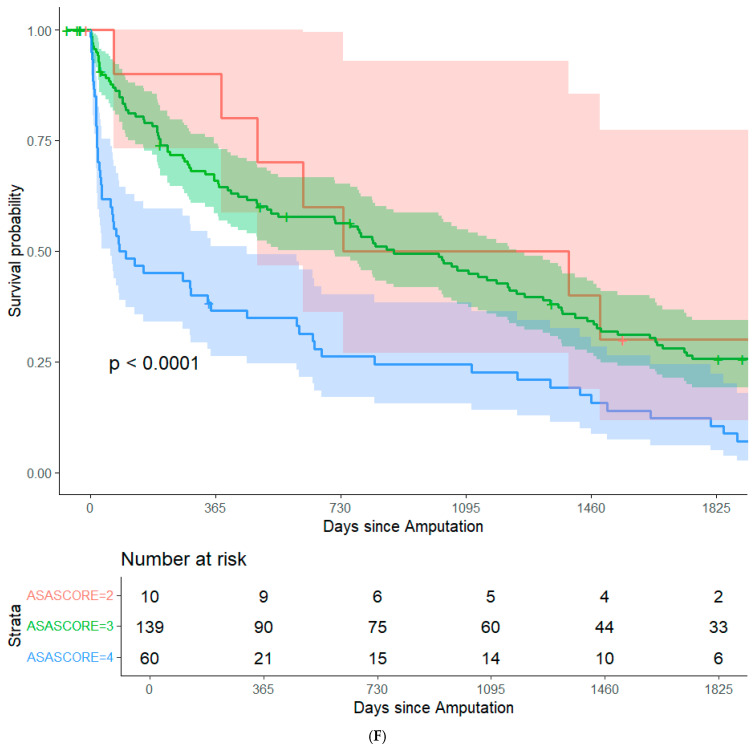
Survival probability estimated by Kaplan–Meier curves and stratified by sex (**A**), history of coronary heart disease (**B**), history of smoking (**C**), surgical re-intervention (**D**), type of major amputation (**E**), and ASA class (**F**). Survival distributions between groups are compared by logrank test, and the associated *p*-values are depicted in the respective figures.

By focusing on data from patients that underwent major amputation between 2012 and 2016, it was feasible to conduct long-term (+5 years) survival analyses while minimizing bias from resulting from right censorship. In summary, 202 patients (82.8%) were recorded in the Austrian National Death Registry. The remaining patients were censored either because they were still alive or because they were lost to follow-up. There were no significant differences in post-operative survival when stratifying the study population based on sex, history of coronary heart disease, history of smoking, surgical re-intervention after amputation, or type of amputation. (Figure 1A–E). Patients with an ASA class of 4 had significantly lower post-operative survival rates than the other patients in the study population (Figure 1F). In an adjusted Cox regression, age at surgery (aHR: 1.03, 95%CI: 1.02–1.05) and ASA class (aHR: 1.61, 95%CI: 1.20–2.15) were both significantly associated with post-operative survival.

### 3.4. Routine Laboratory Parameters After Major Amputation

**Figure 2:**
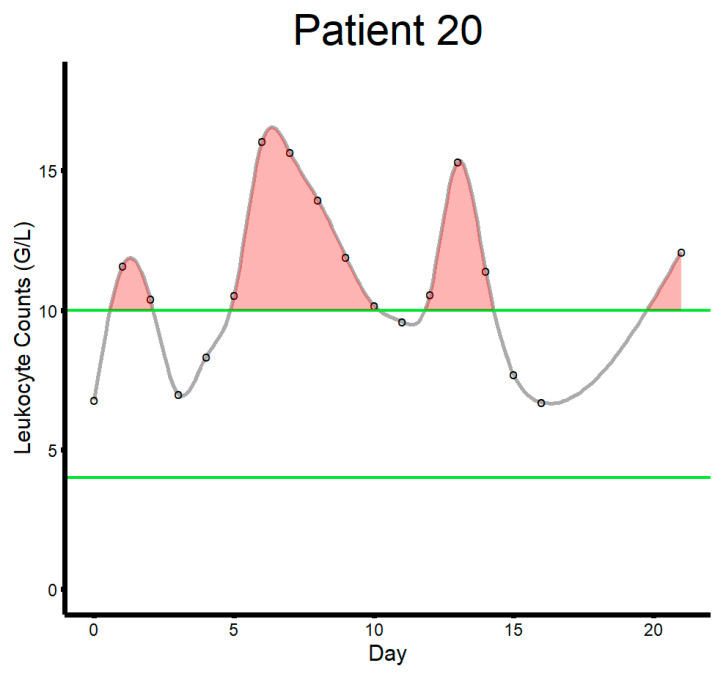
This figure illustrates how the area outside the reference range was calculated for each patient (patient ID 20 in this example). The green lines indicate upper (10.0) and lower bounds (4.0) for leukocyte counts (G/L). The black circles indicate laboratory studies. The gray line indicates the curve connecting laboratory measurements fitting a spline function. The shaded red area illustrates the area above the reference range.

Within 21 days from major amputation, patients in this study population had a mean HGB of 9.5 g/dL and mean leukocyte counts of 10.5 G/L, reflective of the minor state of anemia after major amputation and slightly elevated serological markers of inflammation. Of note, 242 patients (99.2%) had a negative area outside the reference for HGB while 195 patients (79.9%) had a positive area outside the reference for leukocyte counts. Table 2 illustrates metrics derived from laboratory parameters in the study population. Figure 2 illustrates how the area outside the reference was calculated. There were no statistically significant differences between diabetic and non-diabetic patients in any of the calculated laboratory-based metrics.

### 3.5. Laboratory Parameters and Peri-Operative Outcomes

There were no statistically significant differences between the patients who died within the first 30 post-operative days and the remaining patients regarding HGB values besides the area outside the reference. Patients who did not survive the first 30 post-operative days had an area outside the reference of −36 on average, whereas controls had an area outside the reference of −46 on average (*p*-value: 0.013). However, this crude analysis should be interpreted with caution (see below). Regarding leukocyte counts, patients who died in the peri-operative period had higher maximum values (22.3 vs. 15.2, *p*-value: 0.003), higher mean values (16.0 vs. 10.6, *p*-value: < 0.001), and a higher area outside the reference (63 vs. 29, *p*-value: 0.017). Patients who underwent re-intervention had a lower area outside reference regarding HGB (−52 vs. −41, *p*-value: 0.004), a higher area outside the reference regarding leukocyte counts (46 vs. 30, *p*-value: 0.03), higher leukocyte means (12.6 vs. 11.1, *p*-value: 0.032), and higher maximum leukocyte counts (18.4 vs. 15.7, *p*-value: 0.016). However, the crude analyses should be interpreted with caution (see following subsections and Discussion).

### 3.6. Adjusted Survival Analysis Including Laboratory Metrics

After the stepwise selection of laboratory-derived metrics in the Cox regression from the initial survival analysis, it was observed that none of the HGB-related parameters were associated with post-operative survival. However, mean and maximum leukocyte counts as well as the area outside the reference for leukocyte counts were significantly associated after inclusion in the model adjusting for age and ASA class. After including all three, the mean leukocyte count remained statistically significant. Table 3 depicts the model adjusting for age at surgery, ASA class, and mean leukocyte count. Adding the occurrence of a wound complication or sepsis to the model did not relevantly affect the beta coefficient regarding the mean leukocyte count, and both covariates remained below statistical significance. In other words, there is evidence that persistent elevated signs of systemic inflammation are more relevantly associated with long-term mortality than events such as a wound infection or sepsis. In a sensitivity analysis, the workflow was repeated after removing patients who did not survive the peri-operative period of 30 days. ASA class was not significantly associated with survival any longer and was therefore removed from the models. The models with HGB-derived metrics remained constant to the previous step. However, for leukocyte count-associated metrics, only the area outside the reference remained significantly associated with survival (aHR: 1.01, 95%CI: 1.00–1.01) after adjusting for age. In a final sensitivity analysis, it was investigated whether pre-operative HGB or leukocyte counts would be significantly associated with mortality. However, in both cases, the parameter was not significant.

## 4. Discussion

A total of 244 patients who underwent major amputation for peripheral arterial disease (PAD) between 2012 and 2016 were analyzed. This observational period allowed us to investigate long-term mortality but limiting the effects of the global COVID-19 pandemic on the results. Transtibial amputation was performed in 71.3% of cases and transfemoral amputation in 28.7%. The median age was 72 years, with 65.6% of patients being male. Critical limb ischemia at admission was classified as Rutherford stage 4 (18.0%), stage 5 (20.9%), or stage 6 (50.4%), while 10.6% had incomplete staging data. Long-term survival analysis identified 202 patients (82.8%) recorded in the Austrian National Death Registry. Post-operative survival was significantly associated with age (adjusted HR: 1.03, 95% CI: 1.02–1.05) and ASA class (adjusted HR: 1.61, 95% CI: 1.20–2.15). Leukocyte count parameters, including mean values and the area outside the reference, were significantly associated with post-operative survival in adjusted analyses, whereas hemoglobin metrics were not. Sensitivity analysis confirmed leukocyte metrics as predictive factors, particularly after excluding peri-operative deaths.

The findings of this study highlight several key factors influencing post-operative outcomes in patients with PAD undergoing major amputation. The significant association between advanced age and reduced long-term survival aligns with the established literature [17,18,19], emphasizing the impact of age-related comorbidities and frailty on post-operative recovery. Similarly, the strong association between a higher ASA class and poorer survival reflects the influence of pre-existing systemic disease burden on surgical outcomes, which has been frequently shown to be associated with post-operative mortality [20,21,22]. This underscores the importance of optimizing peri-operative care for high-risk patients, potentially through enhanced pre-operative assessments and targeted interventions. The high prevalence of wound-related complications (43.0%) and non-surgical cardiac complications (10.7%) demonstrates the multifactorial risks that these patients face. Notably, wound infections and tissue necrosis may contribute to prolonged hospital stays (median 54 days), increased re-intervention rates (19.6%), and heightened morbidity, emphasizing the need for robust surgical techniques and vigilant post-operative care. The association of leukocyte count metrics with survival further highlights the potential role of systemic inflammation in determining outcomes. Elevated leukocyte levels may reflect an exaggerated inflammatory response, increasing vulnerability to complications and mortality. Events such as a wound infection or even sepsis that are associated with increased leukocyte counts were less informative in our models than the actual mean leukocyte count in the post-operative period. This could indicate that a persistent (low-level) inflammatory state is more relevantly associated with mortality, even in patients who do not experience infectious or inflammatory complications. Conversely, the lack of association between hemoglobin metrics and survival suggests that anemia, while common, may be less predictive in this specific population compared to markers of inflammation.

It is noteworthy that factors such as sex, a history of coronary heart disease, smoking, and amputation type did not significantly influence survival, contrasting with prior research. In the present study population, diabetic patients were also neither more affected by wound complications nor had higher leukocyte-derived metrics. This may indicate that systemic factors, like overall health and inflammatory status, outweigh these individual characteristics in this cohort. These findings advocate for personalized risk stratification models incorporating clinical and laboratory data, particularly inflammatory markers, to guide decision-making. Future research could explore whether interventions targeting inflammation or improving peri-operative support can enhance outcomes for this vulnerable population.

Furthermore, the present analysis should be regarded as a stepping stone for future investigations that leverage a similar workflow in an attempt to propel our understanding of highly prevalent vascular conditions as well as improving patient care and risk stratification. This methodology can both be translated to other pathologies as well as different laboratory parameters that have been linked with post-operative outcomes such as measurements of kidney function or metabolic states.

This study has several limitations that deserve consideration when interpreting the findings. First, its retrospective, single-center design may limit generalizability, as patient characteristics, surgical practices, and post-operative care can vary between institutions. Additionally, the reliance on routinely collected clinical data may introduce inconsistencies or missing information, particularly in cases with incomplete documentation of Rutherford stages or laboratory values. These factors could impact the accuracy of our analyses. Second, while the study identifies significant associations between variables such as age, ASA class, and leukocyte metrics with post-operative survival, these findings do not necessarily reflect a causal relationship. Retrospective observational studies are inherently limited in their ability to disentangle correlation from causation, and unmeasured confounders may influence the results. For example, the association of leukocyte count metrics with survival could reflect an underlying systemic inflammatory state rather than a direct causal relationship and should therefore be considered a surrogate marker. An additional limitation pertains to the calculation of the area outside the reference range for laboratory parameters. Patients with persistent anemia or inflammation are more likely to undergo repeated blood tests, increasing the likelihood of detecting abnormal values and inflating the calculated area outside the reference. This potential bias could exaggerate the observed associations with survival, particularly for patients with more severe or persistent health issues. Care should be taken to interpret these results in the context of clinical complexity and individual variability in monitoring practices. Furthermore, this study assumes that treatment was delivered at the same standard of care in all cases, including adequate antibiotic regimens and blood transfusions in line with guideline recommendations. While treatment, in general, will be delivered based on in-house standard operating procedures and clinical practice guidelines, aberrations thereof might be present in selected cases. Finally, this study did not evaluate the impact of specific post-operative interventions, such as antibiotic regimens or wound care protocols, which could modulate outcomes. Prospective multicenter studies with standardized protocols and advanced statistical techniques to address potential biases are necessary to validate and expand upon these findings.

In conclusion, metrics derived from leukocyte counts extracted from routine laboratory studies can be informative of post-operative mortality risks after major lower extremity amputation. While this might seem intuitive for the peri-operative period, the fact that this also provided information about long-term survival rates after adjusting for relevant confounders is interesting and warrants further investigation to explore whether our findings are generalizable in larger patient cohorts. Identifying which patients might be at an elevated risk of adverse outcomes after surgery is a topic of great interest. Our findings have the potential to be translated into other types of surgery and provide a valuable example that risk assessments might be feasible based on very basic and widely available patient information.

## Figures and Tables

**Table 1:** Patient characteristics of the study population. Censored patients were either alive at the most recently available data extraction from the Austrian Death Registry or lost to follow-up.

Parameter	Censored Patients (n = 42) n (%)	Deceased Patients (n = 202) n (%)	Total (n = 244) n (%)
Age	65 (57–70)	74 (67–80)	72 (65–79)
Body Mass Index (BMI)	26.3 (22.6–29.1)	24.5 (22.2–27.7)	24.7 (22.2–27.8)
Male/Female	29:13	130:72	159:85
Arterial hypertension	26	175	201
Coronary artery disease	15	86	101
Diabetes mellitus	21	127	148
Hyperlipidemia	19	74	93
History of smoking	34	124	158
**ASA class** - 2 - 3 - 4	3 32 4	8 123 58	11 155 62
**Level of amputation** - Transtibial - Transfemoral	33 9	141 61	174 70

**Table 2:** Laboratory parameters used for the analyses given as median (Q1–Q3) (HGB: hemoglobin).

Parameter	Censored Patients (n = 42)	Deceased Patients (n = 202)	Total (n = 244)
Mean HGB	9.7 (9.0–10.7)	9.5 (9.0–10.3)	9.5 (9.0–10.4)
Minimum HGB	8.6 (7.9–9.4)	8.3 (7.5–9.2)	8.4 (7.6–9.3)
Variance HGB	0.7 (0.3–1.3)	0.7 (0.4–1.2)	0.7 (0.4–1.2)
Area outside Reference HGB	−46 (−61–−14)	−45 (−63–−26)	−45 (−62–−26)
Mean Leukocytes	9.9 (7.9–12.5)	10.6 (8.3–13.6)	10.5 (8.1–13.6)
Maximum Leukocytes	14.1 (10.0–19.5)	14.1 (10.9–19.5)	14.2 (10.8–19.5)
Variance Leukocytes	6.0 (2.3–11.5)	6.1 (2.3–14.2)	6.1 (2.3–12.9)
Area outside Reference Leukocytes	8 (0–30)	13 (1–44)	13 (0–43)

**Table 3:** Depicts the Cox model for post-operative mortality in the study population including age at surgery, ASA class, and mean leukocyte counts over the first 21st post-operative days.

Variable	Adjusted Hazard Ratio	95% CI	*p*-Value
Age	1.03	1.02–1.05	<0.001
ASA Class	1.60	1.20–2.13	0.001
Mean Leukocytes	1.09	1.05–1.12	<0.001

## Data Availability

The data that support the findings of this study are available from the corresponding author, upon reasonable request.
